# Sublimable materials facilitate the TEM sample preparation of oil-soluble nanomaterials

**DOI:** 10.1186/s42649-020-00042-7

**Published:** 2020-09-29

**Authors:** Yu-Hao Deng

**Affiliations:** Academy for Advanced Interdisciplinary Studies, Peking University, 209 Chengfu Road, Haidian District, Beijing, 100871 China

**Keywords:** Transmission electron microscopy, Sample preparation, Oil-soluble nanomaterial, Sublimable material

## Abstract

Sample preparation is significantly important to the high-resolution transmission electron microscopy (HRTEM) characterization of nanomaterials. However, many general organic solvents can dissolve the necessary organic polymer support layer in TEM grid, which causes it difficult to obtain high-quality samples of oil-soluble nanomaterials. In this study, a new sample preparation method for oil-soluble nanomaterials has been developed by using the sublimable material as a transition layer. Experiments also show that there is no damage to TEM grids and high-quality HRTEM images can be obtained via this method. This approach paves the way to applicable HRTEM sample preparation of oil-soluble nanomaterials.

## Introduction

**Fig. 1:**
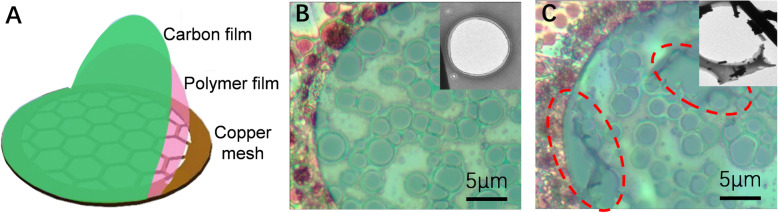
Structure and property of TEM grid. **a** structure of TEM grid for nanomaterials, from top to bottom are carbon film, organic polymer film and copper mesh. **b** optical microscope and TEM images (inset) of unbroken TEM grid. **c** optical microscope and TEM images (inset) destroyed by methylbenzene

Sample preparation is significantly important to the transmission electron microscopy (TEM) characterization for the accurate morphology and crystal structure of nanomaterials (Ayache et al. 2010; Cha et al. 2016; Kim et al. 2014; Park et al. 2016). In order to obtain high-quality high-resolution TEM (HRTEM) images, nanomaterials are often dispersed on the TEM grid, wherein an organic polymer film acts as a reinforced support layer between the ultra-thin carbon film (~ 4 nm) and copper mesh (Fig. 1a) (Regan et al. 2010; Warner et al. 2010; Kennedy et al. 1998). The organic polymer film is necessary for the HRTEM characterization of ultra-small nanomaterials, especially nanocrystalline materials. However, the organic polymer support layer can be dissolved by many general organic reagents (chloroform, methylbenzene, etc), which limits the sample preparation of oil-soluble nanomaterials (Kearns et al. 2006).

## Materials and methods

Methylbenzene (> 99.7%), naphthalene (> 99.9%) were obtained from Sinopharm Chemical Reagent Co., Ltd. (SCRC). TEM grid was obtained from Zhongjingkeyi Technology Co., Ltd. The synthesis of PbS nanocrystals is based on the described by Moreels et al. (2011). The naphthalene is heated to 110 °C and cooled on glass slide to form naphthalene transfer layer. Optical images were acquired using optical microscope (Zeiss Axio Imager, A2m). All TEM, HAADF-STEM and HRTEM images were acquired using Tecnai F30 microscope operated at 300 kV.

## Results and discussion

Figure 1b and c show the TEM grid before and after treatment with methylbenzene respectively. The methylbenzene dissolved the organic polymer film and destroyed the skeleton structure of the TEM grid. The holes damaged by the organic solvent are circled with red dotted lines in Fig. 1c. Uneven and impaired grid will result in a difficult sample searching and sample drift, which will negatively affect the acquisition of high-quality TEM images (Stinson-Bagby et al. 2018; Nair et al. 2010; Duchamp et al. 2014). Therefore, it is necessary to develop a method that can obtain high-quality TEM sample of oil-soluble nanomaterials and avoid damaging the organic polymer layer in TEM grid.

**Fig. 2:**
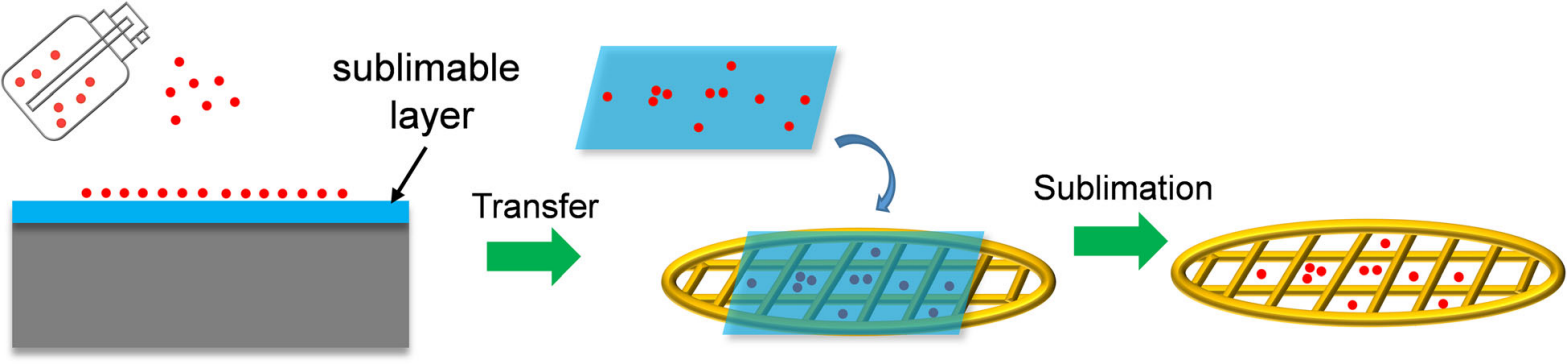
Schematic diagram of TEM sample preparation. Nanomaterials were sprayed onto sublimable layer and transferred on the TEM grid, then place the sample in a vacuum to sublimate naphthalene

Here, a new method for nanomaterial sample preparation was designed by introduce sublimable naphthalene as the transition layer. First, naphthalene was deposited on the substrate and nanomaterials were sprayed onto naphthalene layer. Next, the side of the naphthalene layer with the nanomaterial was transferred to covered on the TEM grid, and then place the sample in a vacuum to sublimate naphthalene. The process is shown in Fig. 2.

**Fig. 3:**
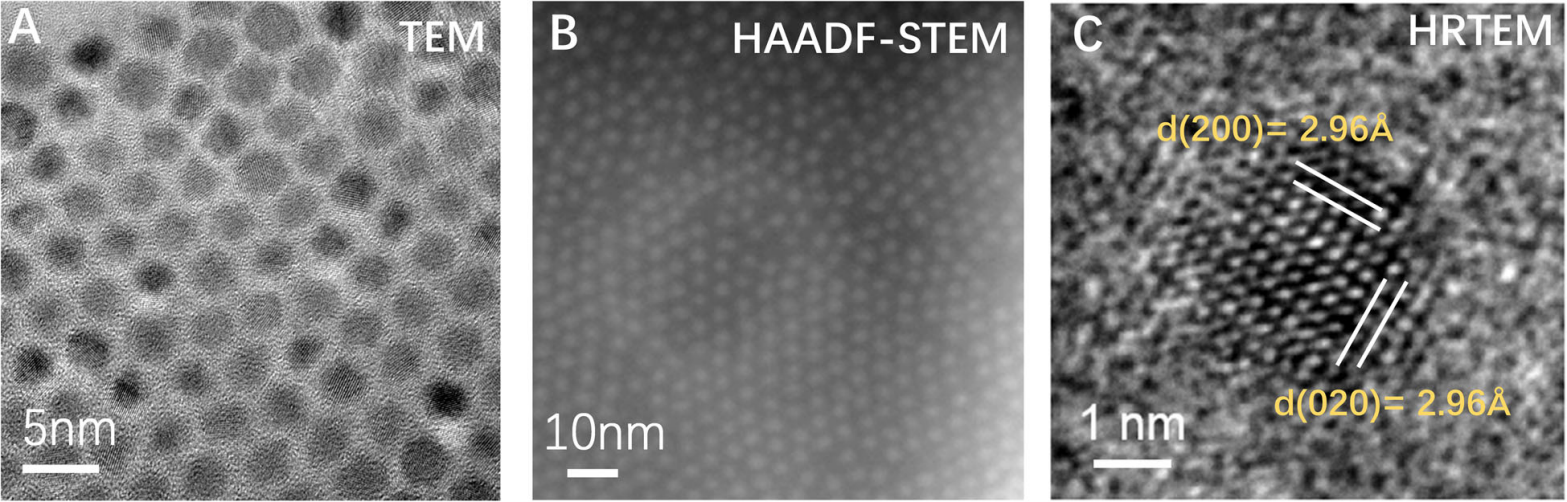
TEM images of PbS nanocrystals sample via new preparation method. **a** TEM image at low magnification. **b** image of nanocrystals under HAADF-STEM mode. **c** image of HRTEM

Based on this method, we obtained a continuous large-scale highly-dispersed oil-soluble PbS nanocrystal sample and captured high-quality TEM (Fig. 3a), High-angle annular dark-field scanning transmission electron microscopy (HAADF-STEM) (Fig. 3b) and HRTEM (Fig. 3c) images. It proves that images with low background contrast, accurate material morphology, and clear lattice fringes can be obtained via this new sample preparation method.

## Conclusions

This new method avoids the destruction of organic polymer layer in TEM grid by organic solvent and can obtain high-quality TEM samples in the characterization of oil-soluble nanomaterials. Our method not only can be used for the preparation of nanocrystals sample, but also can be applied to oil-soluble nanoclusters, nanosheets, nanowires and other materials. In addition to oil-soluble nanomaterials, other nanomaterials in corrosive solutions such as strong acids and bases can also be suitable for this method. This work enriches and improves the sample preparation technique in the field of TEM.

## Data Availability

Please contact the corresponding author for data availability.

## Abbreviations

TEM: Transmission electron microscopy
HRTEM: High resolution transmission electron microscopy
HAADF-STEM: High-angle annular dark-field scanning transmission electron microscopy
PbS: Lead sulfide
